# Enhancing clinical breast examination (CBE) uptake: insights from women in northeastern Peninsular Malaysia

**DOI:** 10.7717/peerj.21029

**Published:** 2026-04-06

**Authors:** Nik Rosmawati Nik Husain, Mohd Khairul Hanan Mohd Wajiah, Nani Draman, Razlina Abdul Rahman, Jamilah Al-Muhammady Mohammad, Mahaneem Mohamed, Wan Zainira Wan Zain

**Affiliations:** 1 Department of Community Medicine, School of Medical Sciences, Health Campus, Universiti Sains Malaysia, Kota Bharu, Kelantan, Malaysia; 2 Department of Family Medicine, School of Medical Sciences, Health Campus, Universiti Sains Malaysia, Kota Bharu, Kelantan, Malaysia; 3 Department of Medical Education, School of Medical Sciences, Health Campus, Universiti Sains Malaysia, Kota Bharu, Kelantan, Malaysia; 4 Department of Physiology, School of Medical Sciences, Health Campus, Universiti Sains Malaysia, Kota Bharu, Kelantan, Malaysia; 5 Department of Surgery, School of Medical Sciences, Health Campus, Universiti Sains Malaysia, Kota Bharu, Kelantan, Malaysia

**Keywords:** Screening uptake, Clinical breast examination, Breast cancer awareness, Healthcare accessibility, Barriers

## Abstract

**Background:**

Clinical breast examination (CBE) is an important component of an early detection strategy for breast cancer in settings where organized screening coverage is limited. This study aimed to determine CBE uptake and its associated factors among women in Kelantan, Northeastern Peninsular Malaysia.

**Methods:**

A cross-sectional study was conducted among 242 women recruited during community health programs across nine districts in Kelantan. Data were collected using a structured questionnaire adapted from the Breast Cancer Awareness Measure Malaysia (BCAM-M). Descriptive analyses summarized participants’ sociodemographic characteristics, breast cancer awareness, and perceived barriers to healthcare-seeking. Multivariable logistic regression was performed among screening-eligible women aged ≥40 years to identify factors associated with CBE uptake.

**Results:**

Among all 242 participants, awareness of breast cancer signs and symptoms was observed in 59.5%, while awareness of breast cancer risk factors (33.5%) and age-related risk (17.4%) was observed in smaller proportions. Perceived barriers to healthcare-seeking were reported by 10.3% of participants. Of the total sample, 112 women (46.3%) were aged ≥40 years and considered screening eligible. Among these women, 72 (64.3%) reported having ever undergone a CBE. Multivariable logistic regression analysis showed that women living in households with ≥6 members had significantly higher odds of undergoing CBE compared with those living in households with 1–5 members (adjusted OR 3.32; 95% CI [1.16–9.49]; *p* = 0.025). Timely healthcare-seeking behaviour was also significantly associated with CBE uptake, with women who sought healthcare within ≤2 months having higher odds of undergoing CBE compared with those who delayed seeking care (adjusted OR 3.78; 95% CI [1.14–12.54]; *p* = 0.030). Education level, income, employment status, marital status, awareness, perceived barriers, and accessibility were not significantly associated with CBE uptake.

**Conclusions:**

Household size and timely healthcare-seeking behaviour were key predictors of CBE uptake among screening-eligible women in Kelantan. Although CBE uptake appeared relatively high, it likely reflects opportunistic or symptom-driven examinations rather than routine screening. Interventions promoting timely healthcare-seeking and leveraging family and social support may help improve early breast cancer detection in settings without organized screening programs.

## Introduction

Breast cancer is a major global health concern, with 2.3 million new cases in 2022, accounting for 11.6% of all cancer diagnoses and making it the second most common cancer worldwide (Bray et al., 2024). It is also the fourth leading cause of cancer-related deaths globally, with approximately 666,000 deaths annually (6.9% of all cancer death) (Bray et al., 2024). In Malaysia, breast cancer is the most common cancer among women, constituting 34.1% of all female cancers, with an age-standardized rate (ASR) of 34.1 per 100,000 (National Cancer Institute Malaysia, 2019). In Northeastern Peninsular Malaysia, the state of Kelantan reports a lower but still significant burden, with breast cancer accounting for 14.4% of female cancers and an ASR of 22.4 per 100,000 (National Cancer Institute Malaysia, 2019). Worryingly, the proportion of Malaysian women presenting with late-stage breast cancer has increased from 43.2% (2007–2011) to 47.9% (2012–2016) (National Cancer Institute Malaysia, 2018).

Early detection through breast cancer screening, facilitates timely diagnosis and treatment and is associated with significant reductions in breast cancer mortality at the population level (Al Hasan, Bennett & Toriola, 2025). Screening methods include clinical breast examination (CBE), ultrasound, and mammography. CBE is recommended every 3 years for women aged 20–39 and annually for those 40 and above by the Ministry of Health Malaysia (2021). CBE is especially relevant in resource-limited settings where access to advanced imaging technologies may be constrained. However, uptake remains low, with national participation at only 26.6% (2016–2020) (Said & Sutan, 2021).

Despite ongoing efforts to raise awareness and promote breast cancer screening, important gaps remain in understanding the factors influencing screening uptake. Previous studies in Malaysia and comparable settings have often examined breast cancer awareness or beliefs in isolation, with limited integration of screening utilization, healthcare-seeking behavior, and accessibility within a single analytical framework (Hasanain et al., 2020; Lee et al., 2019). Furthermore, there is limited research investigating CBE uptake across different age groups and demographic backgrounds, especially in semi-rural settings. These gaps in knowledge hinder the development of targeted interventions to reduce delayed presentation and late-stage breast cancer diagnosis, which are associated with higher mortality as well as substantial economic and psychosocial burdens for affected women and their families.

Addressing these gaps is particularly important in Northeastern Peninsular Malaysia, where contextual factors such as healthcare accessibility, sociodemographic diversity, and healthcare-seeking behavior may influence CBE uptake. By identifying key barriers and facilitators, this study aims to generate evidence to inform culturally appropriate and context-specific public health strategies, strengthen early detection efforts, and ultimately reduce breast cancer-related morbidity and mortality among women in Kelantan.

## Methods

### Study design and participant selection

This cross-sectional study was conducted in Kelantan, a state in Northeastern Peninsular Malaysia, from November 2023 to June 2024. Participants were women aged 18–74 attending community health programs from nine districts in Kelantan. Subjects were recruited using a convenience sampling method. Women with a personal history of breast cancer or any other type of cancer were excluded from the study. The sample size was calculated using Power and Sample Size calculation software for comparing two independent proportions, based on variables from previous studies. The largest required sample size was 220, and after accounting for a 10% dropout rate, the final target sample size was 242.

### Research instrument and data collection

Data were collected using the Breast Cancer Awareness Measure Malaysia (BCAM-M), a validated tool translated from the original UK version into Malay (Htay et al., 2020). The BCAM-M has demonstrated strong internal consistency (Cronbach’s α: 0.73–0.83) and moderate to good test-retest reliability (Intra-class Correlation Coefficient: 0.39–0.69) for awareness, age-related risk, and breast self-examination (BSE) scores. Its content validity was confirmed through expert review, with an Item-Content Validity Index (I-CVI) of 0.83–1.00.

The questionnaire comprised six main domains with 60 items, plus two additional sections. The domains included; (i) Sociodemographic characteristics (10 items: age, ethnicity, education, marital status, occupation, income, *etc*.); (ii) Awareness of breast cancer signs and symptoms (11 items); (iii) Awareness of age-related risk (one item); (iv) Awareness of breast cancer risk factors (10 items on a 5-point Likert scale); (v) Barriers to healthcare-seeking (10 items); and (vi) Breast self-examination (BSE) practice (four items). One additional sections assessing knowledge and experience with CBE (six items) were adapted from a previous study (Mohan et al., 2021).

Data were collected during community health awareness programs. For programs that included health talks or educational exhibitions, questionnaires were administered prior to these activities to minimize information bias. In programs without formal educational components, data collection was conducted throughout the event. Each participant took about 20–25 min to complete the questionnaire, and responses were checked for completeness at the point of collection.

### Ethical approval and consideration

Ethical approval for this study was obtained from the Human Research Ethics Committee (HREC) of Universiti Sains Malaysia (USM), with the approval code USM/JEPeM/KK/23050354, dated 20 August 2023. The study was conducted in accordance with the ethical standards of the 1964 Declaration of Helsinki and its later amendments. Written informed consent was obtained from each participant before their participation. Each participant was provided with a standardized and approved information sheet that explained the details of the study, ensuring their voluntary and informed participation.

### Operational definitions

In this study, participants’ awareness and practices were categorized using specific operational definitions adapted from previous literature. Awareness of breast cancer signs and symptoms was defined as answering “Yes” to at least five questions related to non-lump symptoms (Htay et al., 2020). Awareness of age-related risk was assessed based on whether participants responded “Yes” to the question of whether women aged 50 and above are more likely to develop breast cancer within the next year (Htay et al., 2020). Awareness of breast cancer risk factors and perceived barriers to healthcare-seeking were each classified as scoring ≥60% of the total score in their respective domains (Zhu et al., 2024). Finally, the dependent variable, breast screening uptake, was determined by participants who answered “Yes” to the question, “Has a healthcare provider ever examined your breasts?” (Mohan et al., 2021).

### Data analysis

Statistical analysis was performed using IBM SPSS Statistics for Windows, Version 28.0 (IBM Corp). Descriptive statistics including frequencies and percentages were used to summarize participants’ sociodemographic factors, breast cancer awareness, and perceived barriers. The independent variables included sociodemographic factors (age, marital status, education level, employment status, and household income), individual factors (awareness of breast cancer signs, symptoms, and risk factors), and environmental factors (accessibility to healthcare services). The dependent variable was CBE uptake.

Univariable logistic regression analysis was performed to identify factors associated with CBE uptake. Variables with *p* < 0.25 in univariable analysis and those with epidemiological relevance were included in the multivariable logistic regression model using the forced entry (ENTER) method. Multicollinearity was assessed using variance inflation factors (VIF) and tolerance statistics obtained from linear regression using the same independent variables. A tolerance value < 0.1 or a VIF > 10 was considered indicative of significant multicollinearity. Model fit was assessed using the Hosmer–Lemeshow goodness-of-fit test. Model discrimination was evaluated using the area under the receiver operating characteristic (ROC) curve. Adjusted odds ratios (AOR) with 95% confidence intervals (CI) were reported. Statistical significance was set at *p* < 0.05.

## Results

### Characteristics of participants

The study included 242 women, of whom 130 (53.7%) were aged 18–39 years and 112 (46.3%) were aged ≥40 years. Participants were recruited from nine districts in Kelantan, with the highest representation from Pasir Mas (17.4%), Pasir Puteh (17.4%), and Bachok (17.4%) (Table 1A).

**Table 1:** Sociodemographic characteristics of participants by age group (1A) and by clinical breast examination (CBE) uptake (1B) in northeastern Peninsular Malaysia. Values are presented as frequency and percentage. Participants were categorized into two age groups: 18–39 years and ≥40 years. RM refers to Ringgit Malaysia, the Malaysia national currency. STPM refers to Malaysian higher school certificate.

Variable	Total (*n* = 242)	Age group, *n* (%)	
		18–39 years (*n* = 130)	≥40 years (*n* = 112)
**A. Sociodemographic characteristics of participants by age group (*n* = 242)**			
District			
Bachok	42 (17.4)	13 (10.0)	29 (25.9)
Jeli	25 (10.3)	16 (12.3)	9 (8.0)
Kota Bharu	34 (14.0)	19 (14.6)	15 (13.4)
Kuala Krai	22 (9.1)	12 (9.2)	10 (8.9)
Machang	10 (4.1)	7 (5.4)	3 (2.7)
Pasir Mas	43 (17.4)	19 (14.6)	24 (21.4)
Pasir Puteh	42 (17.4)	25 (19.2)	17 (15.2)
Tanah Merah	3 (1.2)	1 (0.8)	2 (1.8)
Tumpat	21 (8.7)	18 (13.8)	3 (2.7)
Marital status			
Single/Divorced/Widow	65 (26.9)	43 (33.1)	22 (19.6)
Married	177 (73.1)	87 (66.9)	90 (80.4)
Education level			
Primary/secondary	95 (39.3)	39 (30.0)	56 (50.0)
STPM^a^/Diploma and above	147 (60.7)	91 (70.0)	56 (50.0)
Employment status			
Housewife/Student/unemployed/retiree	91 (37.6)	52 (40.0)	39 (34.8)
Government sector	87 (36.0)	42 (32.3)	45 (40.2)
Non-government sector	64 (26.4)	36 (27.7)	28 (25.0)
Household size			
1–5	156 (64.5)	84 (64.6)	72 (64.3)
≥6	86 (35.5)	46 (35.4)	40 (35.7)
Household income			
No fixed income/<RM^b^ 3000	153 (63.2)	89 (68.5)	64 (57.1)
≥RM 3000	89 (36.8)	41 (31.5)	48 (42.9)
Accessibility to a healthcare facility			
>5 km	48 (19.8)	28 (21.5)	20 (17.9)
≤5 km	194 (80.2)	102 (78.5)	92 (82.1)

Variable	Total ≥40 years (*n* = 112)	CBE^c^ uptake, *n* (%)	
		No (*n* = 40)	Yes (*n* = 72)
**B. Sociodemographic characteristics of women aged ≥40 years by clinical breast examination uptake (*n* = 112)**			
District			
Bachok	29 (25.9)	10 (25.0)	19 (26.4)
Jeli	9 (8.0)	3 (7.5)	6 (8.3)
Kota Bharu	15 (13.4)	4 (10.0)	11 (15.3)
Kuala Krai	10 (8.9)	3 (7.5)	7 (9.7)
Machang	3 (2.7)	1 (2.5)	2 (2.8)
Pasir Mas	24 (21.4)	11 (27.5)	13 (18.1)
Pasir Puteh	17 (15.2)	3 (7.5)	14 (19.4)
Tanah Merah	2 (1.8)	2 (5.0)	0 (0.0)
Tumpat	3 (2.7)	3 (7.5)	0 (0.0)
Marital status			
Single/Divorced/Widow	22 (19.6)	7 (17.5)	15 (20.8)
Married	90 (80.4)	33 (82.5)	57 (79.2)
Education level			
Primary/secondary	56 (50.0)	26 (65.0)	30 (41.7)
STPM^a^/Diploma and above	56 (50.0)	14 (35.0)	42 (58.3)
Employment status			
Housewife/Student/unemployed/retiree	39 (34.8)	18 (45.0)	21 (29.2)
Government sector	45 (40.2)	10 (25.0)	35 (48.6)
Non-government sector	28 (25.0)	12 (30.0)	16 (22.2)
Household size			
1–5	72 (64.3)	31 (77.5)	41 (56.9)
≥6	40 (35.7)	9 (22.5)	31 (43.1)
Household income			
No fixed income/<RM^b^ 3000	64 (57.1)	28 (70.0)	36 (50.0)
≥RM 3000	48 (42.9)	12 (30.0)	36 (50.0)
Accessibility to a healthcare facility			
>5 km	20 (17.9)	8 (20.0)	12 (16.7)
≤5 km	92 (82.1)	32 (80.0)	60 (83.3)

**Notes:**

a STPM = Malaysian higher school certificate.

b RM = Ringgit Malaysia (Malaysian currency).

c CBE = Clinical breast examination.

Overall, the majority of participants were married (73.1%) and had a household income of less than RM 3000 (63.2%). Most participants had attained STPM, diploma, or higher educational qualifications (60.7%). Government sector employees constituted the largest employment group (36.0%), followed by housewives, students, unemployed, or retirees (37.6%). Most participants lived in households with one to five members (64.5%), and the majority (80.2%) resided within five kilometers of a healthcare facility.

Among women aged ≥40 years (screening-eligible group), 72 (64.3%) reported having ever undergone a CBE, while 40 (35.7%) reported no prior CBE (Table 1B). Within this age group, women who reported CBE uptake were more commonly married (79.2%), employed in the government sector (48.6%), and living within five kilometers of a healthcare facility (83.3%). Detailed sociodemographic characteristics stratified by age group and CBE uptake are presented in Tables 1A and 1B.

### Breast cancer awareness and perceived barriers

Awareness of breast cancer signs and symptoms was observed in 59.5% of participants. In comparison, awareness of breast cancer risk factors (33.5%) and age-related breast cancer risk (17.4%) was identified in a smaller proportion of participants (Table 2). A lump in the breast was the most recognized symptom (83.1%), while nipple rash was the least recognized (40.5%) (Table 3). The history of breast cancer was the most acknowledged risk factor (61.2% agreed or strongly agreed), whereas having a late menopause was the least known (21.5% agreed or strongly agreed) (Table 4). Perceived barriers to healthcare-seeking were identified in 10.3% of participants. Emotional barriers were the most prominent, with embarrassment (46.7%), fear of diagnosis (40.9%), and fear of doctors (39.6%) being the major concerns (Table 5).

**Table 2:** Proportion of breast cancer awareness and perceived barriers to healthcare-seeking among participants in northeastern Peninsular Malaysia (*n* = 242). Values represent the number and percentage of participants meeting predefined criteria for awareness of breast cancer signs and symptoms, awareness of age-related risk, awareness of breast cancer risk factors, and perceived barriers to healthcare-seeking.

Variable	*n* (%)
Awareness of breast cancer signs and symptoms	144 (59.5)
Awareness of age-related risk	42 (17.4)
Awareness of breast cancer risk factors	81 (33.5)
Perceived barriers to healthcare-seeking	25 (10.3)

**Table 3:** Awareness of breast cancer signs and symptoms among participants in northeastern Peninsular Malaysia (*n* = 242). Values are presented as frequency and percentage of participants who responded “Yes,” “No,” “Do not know,” or did not respond to each item assessing awareness of breast cancer signs and symptoms.

Item	Yes	No	No answer	Do not know
	*n* (%)			
Change in nipple position	118 (48.8)	72 (29.7)	5 (2.1)	47 (19.4)
Nipple retraction	117 (48.3)	66 (27.3)	8 (3.3)	51 (21.1)
Pain in one of the breast or armpit	197 (81.4)	29 (12.0)	1 (0.4)	15 (6.2)
Puckering or dimpling of breast skin	115 (47.5)	60 (24.8)	6 (2.5)	61 (25.2)
Discharge or bleeding from nipple	184 (76.0)	25 (10.3)	1 (0.4)	32 (13.3)
Lump or thickening in the breast	201 (83.1)	14 (5.8)	5 (2.1)	22 (9.0)
Nipple rash	98 (40.5)	75 (31.0)	8 (3.3)	61 (25.2)
Redness of the breast skin	113 (46.7)	64 (26.4)	6 (2.5)	59 (24.4)
Lump or thickening in the armpit	181 (74.8)	24 (9.9)	3 (1.2)	34 (14.1)
Changes in the size of the breast or nipple	147 (60.8)	48 (19.8)	0 (0.0)	47 (19.4)
Changes in the shape of breast or nipple	152 (62.8)	35 (14.5)	6 (2.5)	49 (20.2)

**Table 4:** Awareness of breast cancer risk factors among participants in northeastern Peninsular Malaysia (*n* = 242). Values are presented as frequency and percentage according to participants’ level of agreement with each breast cancer risk factor using a Likert scale. HRT refers to hormone replacement therapy. OCP refers to oral contraceptive pills. Overweight refers to body mass index ≥25 kg/m².

Item	Strongly disagree	Disagree	Not sure	Agree	Strongly agree	No reply
	*n* (%)					
Past history of breast cancer	17 (7.0)	20 (8.3)	34 (14.0)	114 (47.1)	34 (14.1)	23 (9.5)
Using HRT^a^	9 (3.7)	20 (8.3)	94 (38.8)	76 (31.4)	12 (5.0)	31 (12.8)
Using OCP^b^	9 (5.8)	29 (12.0)	93 (38.4)	66 (27.3)	12 (5.0)	33 (13.6)
Drinking >1 unit of alcohol/day	14 (5.8)	28 (11.6)	83 (34.3)	76 (31.4)	15 (6.2)	26 (10.7)
Being overweight^c^	8 (3.3)	34 (14.0)	97 (40.1)	61 (25.2)	15 (6.2)	27 (11.2)
Having a close relative with breast cancer	12 (5.0)	25 (10.3)	40 (16.5)	106 (43.8)	33 (13.6)	26 (10.8)
Having children later in life or not at all	10 (4.1)	36 (14.9)	84 (34.7)	73 (30.2)	8 (3.3)	31 (12.8)
Starting periods at an early age	14 (5.8)	45 (18.6)	102 (42.1)	49 (20.2)	6 (2.5)	26 (10.7)
Having late menopause	12 (5.0)	41 (16.9)	107 (44.2)	48 (19.8)	4 (1.7)	30 (12.4)
Insufficient moderate physical activity (≤30 min, 5 days/week)	24 (9.9)	48 (19.8)	83 (34.3)	52 (21.5)	9 (3.7)	26 (10.7)

**Notes:**

a Hormone replacement therapy.

b Oral contraceptive pill.

c Body mass index ≥ 25 kg/m^2^.

**Table 5:** Perceived barriers to healthcare-seeking for breast-related symptoms among participants in northeastern Peninsular Malaysia (*n* = 242). Values are presented as frequency and percentage of participants reporting barriers as “Yes, often,” “Yes, sometimes,” “No,” “Do not know,” or no response.

Item	No	Yes, often	Yes, sometimes	No reply	Do not know
	*n* (%)				
Too embarrassed to go and see the doctor	124 (51.2)	52 (21.5)	61 (25.2)	4 (1.7)	1 (0.4)
Too scared to go and see the doctor	142 (58.8)	48 (19.8)	48 (19.8)	3 (1.2)	1 (0.4)
Worried about wasting the doctor’s time	208 (86.0)	8 (3.3)	14 (5.8)	10 (4.1)	2 (0.8)
I find my doctor difficult to talk to	195 (80.6)	17 (7.0)	22 (9.1)	6 (2.5)	2 (0.8)
Difficult to make an appointment with the doctor	175 (72.3)	20 (8.3)	36 (14.9)	8 (3.3)	3 (1.2)
Too busy to make time to go to the doctor	137 (56.6)	31 (12.8)	64 (26.5)	7 (2.9)	3 (1.2)
Too many other things to worry about	145 (59.9)	35 (14.5)	50 (20.6)	8 (3.3)	4 (1.7)
Difficult to arrange transport to the clinic	197 (81.4)	15 (6.2)	25 (10.3)	4 (1.7)	1 (0.4)
Worrying about what the doctor might find	136 (56.2)	51 (21.1)	48 (19.8)	4 (1.7)	3 (1.2)
Not feeling confident talking about the symptoms with the doctor	167 (69.0)	28 (11.6)	36 (14.9)	9 (3.7)	2 (0.8)

### Factors associated with clinical breast examination uptake

The multivariable logistic regression analysis was conducted among women aged ≥40 years, for whom CBE is recommended (Table 6). After adjustment for potential confounders, two factors were independently associated with CBE uptake.

**Table 6:** Factors associated with clinical breast examination uptake among women aged ≥40 years in northeastern Peninsular Malaysia: univariable and multivariable logistic regression analysis (*n* = 112). Crude and adjusted odds ratios (OR) with 95% confidence intervals (CI) were estimated using logistic regression to identify factors associated with clinical breast examination uptake. Variables with *p* < 0.25 in univariable analysis and epidemiological relevance were included in the multivariable model using the ENTER method. Ref indicates reference category. STPM refers to Malaysian higher school certificate.

Variable	Wald statistics (df)	Crude OR (95% CI)	*p*-value^a^	Adjusted OR (95% CI)	*p*-value^b^
Marital status					
Single/Divorced/Widow		Ref			
Married	0.18 (1)	0.81 [0.30–2.18]	0.671		
Education level					
Primary/secondary		Ref			
STPM^c^/Diploma and above	5.47 (1)	2.60 [1.17–5.79]	0.019		
Employment status					
Housewife/Student/unemployed/retiree		Ref			
Government sector	5.21 (1)	3.00 [1.17–7.71]	0.022		
Non-government sector	0.07 (1)	1.14 [0.43–3.04]	0.789		
Household size					
1–5		Ref			
≥6	4.58 (1)	2.60 [1.08–6.26]	0.032	3.32 [1.16–9.49]	0.025
Household income					
No fixed income and ≤RM 3000		Ref			
≥RM 3000	4.11 (1)	2.33 [1.03–5.29]	0.043		
Duration for healthcare-seeking					
>2 months		Ref			
≤2 months	7.47 (1)	3.36 [1.41–7.99]	0.006	3.78 [1.14–12.54]	0.030
Awareness of breast cancer signs and symptoms					
No		Ref			
Yes	1.14 (1)	1.54 [0.70–3.39]	0.285		
Awareness of age-related risk					
No		Ref			
Yes	0.35 (1)	1.37 [0.48–3.90]	0.557		
Awareness of breast cancer risk factors					
No		Ref			
Yes	0.43 (1)	1.32 [0.58–3.02]	0.513		
Perceived barriers to healthcare-seeking					
No		Ref			
Yes	2.62 (1)	3.00 [0.79–11.35]	0.106		
Accessibility to a healthcare facility					
>5 km		Ref			
≤5 km	0.19 (1)	1.25 [0.46–3.37]	0.659		

**Notes:**

a Univariable logistic regression.

b Multivariable logistic regression.

c STPM = Malaysian higher school certificate.

OR, Odds ratio; CI, confidence interval; Ref, reference category.

District was excluded from multivariable analysis due to sparse data and zero cell counts.

Variables with *p* < 0.25 in univariable logistic regression and epidemiological relevance were included in the multivariable model.

ENTER method was applied.

No evidence of collinearity.

Hosmer-Lemeshow test, *p*-value = 0.484.

Area under receiver operating characteristics (ROC) curve was 77.6%.

Women living in households with six or more members had higher odds of CBE uptake (Adjusted OR = 3.32; 95% CI [1.16–9.49]; *p* = 0.025), compared with those living in households with one to five members. Healthcare-seeking behaviour was also significantly associated with CBE uptake. Women who sought healthcare within 2 months of noticing breast-related symptoms had higher odds of undergoing CBE compared with those who delayed seeking care (Adjusted OR 3.78; 95% CI [1.14–12.54]; *p* = 0.030).

Other variables, including marital status, educational level, employment status, household income, awareness of breast cancer signs and symptoms, awareness of breast cancer risk factors, perceived barriers to healthcare-seeking, and accessibility to healthcare facilities, were not independently associated with CBE uptake after adjustment. Refer to Table 6.

## Discussion

This study examined factors associated with clinical breast examination (CBE) uptake among women in Kelantan, Malaysia, focusing on those aged ≥40 years who are eligible for screening. The findings showed that household size and timely healthcare-seeking behaviour were significantly associated with CBE uptake. Women living in larger households and those who sought healthcare promptly had higher odds of undergoing CBE. In contrast, sociodemographic characteristics, awareness-related variables, perceived barriers, and accessibility to healthcare facilities were not independently associated with CBE uptake after adjustment.

### Breast cancer awareness

The study found that awareness of breast cancer signs and symptoms was observed in 59.5% of women, whereas awareness of breast cancer risk factors (33.5%) and age-related risk (17.4%) was observed in a substantially smaller proportion of participants. These figures are lower than those reported in studies from other regions, such as China (Zhu et al., 2024). This gap in knowledge may be linked to the socioeconomic profile of the participants, a majority of whom belonged to lower-income households. Lower socioeconomic status often correlates with limited access to quality education and health resources, which in turn affects health literacy (Al-Hanawi et al., 2020; Svendsen et al., 2020). Limited access to digital resources like the internet, which can be a vital source of health information, may further exacerbate this knowledge (Li et al., 2020). This lack of comprehensive awareness can lead to a low perception of personal risk, delaying preventive actions and contributing to late-stage diagnoses.

### Perceived barriers to healthcare seeking

Although only 10.3% of participants were classified as having perceived barriers, emotional barriers were the most frequently cited obstacles. Embarrassment (46.7%), fear of a cancer diagnosis (40.9%), and fear of doctors (39.6%) were prominent, consistent with findings from previous studies in Malaysia (Mohan et al., 2021; Su et al., 2020). These psychological barriers can be powerful deterrents, often overriding knowledge about the importance of early detection (Rajaram et al., 2023). Cultural taboos surrounding discussions of breast health in some Malaysian communities may amplify these feelings of fear and embarrassment, creating a silent barrier to seeking care (Schliemann et al., 2019). Addressing these emotional and cultural factors is essential for improving CBE uptake.

### Clinical breast examination uptake in the screening-eligible population

Nearly two-thirds of women aged ≥40 years reported having ever undergone a CBE. This level of uptake is higher than figures reported in national and regional studies in Malaysia, where CBE utilization has generally ranged between 25% and 36% (Mohan et al., 2021; Said & Sutan, 2021). This estimate should be interpreted within the context of the study’s operational definition and sampling approach, as CBE was defined as a lifetime examination and may reflect a combination of opportunistic, diagnostic, and preventive encounters rather than guideline-adherent routine screening (Ministry of Health Malaysia, 2021). In addition, recruitment through community health programs may have contributed to an overestimation of uptake, as participants attending such programs tend to be more health-conscious and have higher health literacy than the general population (Baccolini et al., 2022).

### Factors associated with breast screening uptake

#### Household size

Household size was significantly associated with CBE uptake, with women living in households with six or more members had higher odds of having ever undergone CBE compared with those living in smaller households (one to five members). This finding contrasts with some previous studies that reported larger household size as a barrier to breast cancer screening, often due to competing caregiving responsibilities and limited resources (Sun et al., 2022).

A plausible explanation for the positive association observed in this study is the role of family and social support in facilitating healthcare engagement. Larger households may provide interpersonal encouragement, shared health information, and practical assistance, such as accompaniment to healthcare facilities, which can reduce barriers to preventive care. Consistent with this explanation, higher levels of social support have been associated with increased participation in breast cancer screening, while lower social support has been linked to non-participations (Jensen et al., 2015; Pohl et al., 2025). These findings suggest that the influence of household size on screening behavior is context-dependent and may function as a facilitator in settings where family support is strong.

#### Duration of healthcare-seeking

This study found that the duration of healthcare-seeking was significantly associated with breast screening uptake, with women who sought medical care within 2 months having higher odds of undergoing CBE compared with those who delayed care for more than 2 months. This finding aligns with health behavior theory, which suggests that individuals with greater health knowledge, higher perceived susceptibility and severity, and stronger perceived benefits are more likely to engage in timely healthcare-seeking behaviors (Kussia et al., 2024; Rosenstock, 1974).

Women who seek healthcare promptly are also more likely to possess higher health literacy, enabling them to recognize breast cancer risks, understand the importance of early detection, and engage in screening-related procedures such as CBE (Levy & Janke, 2016). Healthcare-seeking behavior, however, is shaped by multiple interacting factors. According to the behavioral model of health service utilization, healthcare use is influenced by predisposing factors (*e.g*., age, education, health beliefs), enabling factors (*e.g*., income and access to services), and need factors (*e.g*., perceived symptoms and illness severity) (Chomi et al., 2014).

### Non-significant sociodemographic and awareness factors

In this study, education level, household income, employment status, marital status, accessibility to healthcare facilities, and awareness-related variables were not independently associated with CBE uptake after adjustment. These findings are consistent with previous studies showing that sociodemographic characteristics may lose significance after accounting for behavioural and healthcare engagement factors (Mohan et al., 2021; Ngan et al., 2022).

Education and income are known to influence screening indirectly through health literacy and healthcare engagement rather than acting as direct determinants (Jansen et al., 2018). Similarly, awareness alone may not be sufficient to drive screening participation, as behavioural readiness, healthcare-seeking practices, and provider interaction play more immediate roles in influencing screening uptake (Abdullah et al., 2022; Rajaram et al., 2023).

Accessibility to healthcare facilities was also not associated with CBE uptake, likely reflecting the relatively good geographic access in this study population. When access barriers are minimal, behavioural and social factors may have greater influence on screening participation than structural factors alone (Su & Donnelly, 2022).

## Strengths and limitations

This study has several strengths. First, it provides context-specific evidence on CBE uptake in Northeastern Peninsular Malaysia, a semi-rural setting that is underrepresented in breast cancer screening research. Second, data were collected using the validated Breast Cancer Awareness Measure Malaysia (BCAM-M), ensuring acceptable reliability and content validity of awareness and barrier constructs. Third, participants were recruited from nine districts, enhancing geographical coverage and allowing exploration of district-level variation.

However, this study is not without limitations. First, the cross-sectional design precludes causal inference between the identified factors and CBE uptake. Second, data collection conducted during community health programs using convenience sampling may have introduced selection bias, as participants were likely more health-conscious and health-literate than the general population, potentially leading to an overestimation of CBE uptake. Third, CBE uptake was operationalized as having ever undergone CBE, which does not distinguish between preventive, opportunistic, or diagnostic examinations, nor does it capture adherence to guideline-recommended screening intervals. Fourth, reliance on self-reported data may have resulted in recall bias and social desirability bias. Finally, the relatively small sample size in certain subgroups may have limited statistical power to detect associations for some variables.

## Conclusions

In conclusion, this study demonstrates that household size, and timely healthcare-seeking behavior are significant predictors of CBE uptake among screening-eligible women in Kelantan. Although the observed CBE uptake among women aged ≥40 years was relatively high, this finding should be interpreted within the context of the study design and sampling approach, as it likely reflects a combination of opportunistic and symptom-driven examinations rather than routine, guideline-adherent screening. Persistently low awareness of age-related breast cancer risk and breast cancer risk factors indicate ongoing gaps in breast health knowledge.

Collectively, these findings underscore the importance of leveraging family and social support structures and encouraging prompt help-seeking to improve early detection. By addressing these key determinants, it is possible to enhance early detection rates, improve survival outcomes, and reduce the burden of breast cancer in northeastern Peninsular Malaysia.

## Supplemental Information

10.7717/peerj.21029/supp-1 Supplemental Information 1 STROBE checklist.

10.7717/peerj.21029/supp-2 Supplemental Information 2 Questionnaire: Breast Cancer Awareness Measure Malaysia (BCAM-M), English.

10.7717/peerj.21029/supp-3 Supplemental Information 3 Questionnaire: Breast Cancer Awareness Measure Malaysia (BCAM-M), Malay.

10.7717/peerj.21029/supp-4 Supplemental Information 4 Codebook for dataset for SPSS.

10.7717/peerj.21029/supp-5 Supplemental Information 5 Data.

## Additional Information and Declarations

### Data Availability

The following information was supplied regarding data availability:

The data is available in the Supplemental File.
