# Supplementary material for: Enhancing clinical breast examination (CBE) uptake: insights from women in northeastern Peninsular Malaysia
Source: PeerJ. 2026 Apr 6;14:e21029. doi: 10.7717/peerj.21029 (PMC13064672; doi:10.7717/peerj.21029)
Supplement: Supplemental Information 1 [file peerj-14-21029-s001.doc]

STROBE Statement—Checklist of items that should be included in reports of ***cross-sectional studies***

|  | Item No | Recommendation |  | Check |  |
| --- | --- | --- | --- | --- | --- |
| **Title and abstract** | 1 | (*a*) Indicate the study’s design with a commonly used term in the title or the abstract |  | Study design stated as 'cross-sectional study' (Abstract-Line 31, Methods-Line 87). |  |
|  |  | (*b*) Provide in the abstract an informative and balanced summary of what was done and what was found |  | **Abstract** – Background, Methods, Results, Conclusions. |  |
| Introduction | | |  |  |  |
| Background/rationale | 2 | Explain the scientific background and rationale for the investigation being reported |  | **Introduction** – Line 49-59 Rationale-Introduction-Line 69-76 |  |
| Objectives | 3 | State specific objectives, including any prespecified hypotheses |  | **Introduction** – Line 78-83 |  |
| Methods | | |  |  |  |
| Study design | 4 | Present key elements of study design early in the paper |  | **Methods** – 'Study design and participant selection'-Line 87 |  |
| Setting | 5 | Describe the setting, locations, and relevant dates, including periods of recruitment, exposure, follow-up, and data collection |  | **Methods** – Kelantan, Nov 2023–Jun 2024. Line 88 |  |
| Participants | 6 | (*a*) Give the eligibility criteria, and the sources and methods of selection of participants |  | **Methods** – 'Study design and participant selection'-Line 88-91. |  |
| Variables | 7 | Clearly define all outcomes, exposures, predictors, potential confounders, and effect modifiers. Give diagnostic criteria, if applicable |  | **Methods** – Operational definitions. Line 129-140 |  |
| Data sources/ measurement | 8* | For each variable of interest, give sources of data and details of methods of assessment (measurement). Describe comparability of assessment methods if there is more than one group |  | **Methods** – 'Research instrument and data collection'. Line 97-110 |  |
| Bias | 9 | Describe any efforts to address potential sources of bias |  | **Method-**Line 112**, Discussion** – Strengths and limitations section.Line 285-290 |  |
| Study size | 10 | Explain how the study size was arrived at |  | **Methods** – Line 91-94 sample size calculation |  |
| Quantitative variables | 11 | Explain how quantitative variables were handled in the analyses. If applicable, describe which groupings were chosen and why |  | **Methods** – Data analysis section. Line 144- Descriptive analysis  Line 151-153- Simple and Multiple Logistic regression |  |
| Statistical methods | 12 | (*a*) Describe all statistical methods, including those used to control for confounding |  | **Methods –** Line 151-154- Simple and Multiple Logistic regression |  |
|  |  | (*b*) Describe any methods used to examine subgroups and interactions |  | Data Analysis – Line 153-157 |  |
|  |  | (*c*) Explain how missing data were addressed |  | **Method-**Research instrument and Data collection. Line 114-116 |  |
|  |  | (*d*) If applicable, describe analytical methods taking account of sampling strategy |  | **Methods** – convenience sampling explained. Line 90 |  |
|  |  | (*e*) Describe any sensitivity analyses |  | Not applicable – not performed as the data were complete and logistic regression assumption were met |  |
| Results | | |  |  |  |
| Participants | 13* | (a) Report numbers of individuals at each stage of study—eg numbers potentially eligible, examined for eligibility, confirmed eligible, included in the study, completing follow-up, and analysed |  | **Results** – Characteristics of participants, Table 1. |  |
|  |  | (b) Give reasons for non-participation at each stage |  | Not applicable – convenience sample. |  |
|  |  | (c) Consider use of a flow diagram |  | Not applicable – no flow diagram used. |  |
| Descriptive data | 14* | (a) Give characteristics of study participants (eg demographic, clinical, social) and information on exposures and potential confounders |  | **Results** – Characteristics of participants .Table 1. |  |
|  |  | (b) Indicate number of participants with missing data for each variable of interest |  | **Method**-No missing data. Each Responses were checked for completeness before concluding data collection. Line 114-116 |  |
| Outcome data | 15* | Report numbers of outcome events or summary measures |  | **Results** – Breast Self-examination (BSE) and Clinical Breast examination (CBE) uptake Line 188-190 |  |
| Main results | 16 | (*a*) Give unadjusted estimates and, if applicable, confounder-adjusted estimates and their precision (eg, 95% confidence interval). Make clear which confounders were adjusted for and why they were included |  | **Results** – Table 7, Multiple logistic regression. |  |
|  |  | (*b*) Report category boundaries when continuous variables were categorized |  | **Results** – Table 1 |  |
|  |  | (*c*) If relevant, consider translating estimates of relative risk into absolute risk for a meaningful time period |  | Not applicable – not performed. |  |
| Other analyses | 17 | Report other analyses done—eg analyses of subgroups and interactions, and sensitivity analyses |  | **Results** – factors associated with CBE uptake. Subgroup and interaction check ( not significant (sensitivity analysis not performed) |  |
| Discussion | | |  |  |  |
| Key results | 18 | Summarise key results with reference to study objectives |  | **Discussion** – first paragraph. Line 207-212 |  |
| Limitations | 19 | Discuss limitations of the study, taking into account sources of potential bias or imprecision. Discuss both direction and magnitude of any potential bias |  | **Discussion** – Strengths and limitations section. Line 289-297 |  |
| Interpretation | 20 | Give a cautious overall interpretation of results considering objectives, limitations, multiplicity of analyses, results from similar studies, and other relevant evidence |  | **Discussion** – Line 207-286 |  |
| Generalisability | 21 | Discuss the generalisability (external validity) of the study results |  | **Discussion** – Strengths and limitations section. Line 289-297 |  |
| Other information | | |  |  |  |
| Funding | 22 | Give the source of funding and the role of the funders for the present study and, if applicable, for the original study on which the present article is based |  | **Funding section** – Kelantan State Government grant. Linne 322 |  |

*Give information separately for exposed and unexposed groups.

**Note:** An Explanation and Elaboration article discusses each checklist item and gives methodological background and published examples of transparent reporting. The STROBE checklist is best used in conjunction with this article (freely available on the Web sites of PLoS Medicine at http://www.plosmedicine.org/, Annals of Internal Medicine at http://www.annals.org/, and Epidemiology at http://www.epidem.com/). Information on the STROBE Initiative is available at www.strobe-statement.org.
