# Supplementary material for: Enhancing clinical breast examination (CBE) uptake: insights from women in northeastern Peninsular Malaysia
Source: PeerJ. 2026 Apr 6;14:e21029. doi: 10.7717/peerj.21029 (PMC13064672; doi:10.7717/peerj.21029)
Supplement: Supplemental Information 2 [file peerj-14-21029-s002.pdf]

Serial No.:______

Breast Cancer Awareness and Factors Related to Breast Self-Examination and Acceptance of Breast Screening Among Women in Kelantan

QUESTIONNAIRE FORM

This questionnaire consists of six sections (A – F) as follows:

Section A	Sociodemographic information	9 questions (1-9)
Section B	Knowledge about breast cancer	11 questions (1-21)
Section C	Breast self-examination	4 questions (22-25)
Section D	Barriers to breast cancer detection	11 questions (26-36)
Section E	Knowledge about breast cancer risks	12 questions (37-48)
Section F	Clinical breast examination	1 questions (49 a-f)

A. SOCIODEMOGRAPHIC INFORMATION

This section aims to gather information about your demographics.
All questions must be answered. Please tick **(✓)** in the appropriate space.

1a. What is your citizenship?
( ) Malaysia ( ) Non-Malaysia
( ) No answer ( ) Don't know

1b. Age: [ ] years

2. What is your race?
( ) Malay ( ) Chinese
( ) Indian ( ) Other Bumiputra
( ) No answer ( ) Don't know
( ) Others (Specify) : ____________

3. What is your religion?
( ) Islam ( ) Christian
( ) Buddhist ( ) Hindu
( ) Sikh ( ) Don't know
( ) No answer
( ) Others (Specify) : ____________

4. What is your marital status?
( ) Single ( ) Divorced
( ) Married ( ) Widow/Widower
( ) No answer ( ) Don't know

5. How many people are in your household?? ______ people
(Everyone living in the house, including children)
( ) No answer ( ) Don't know

6. What is your highest level of education?
( ) No schooling ( ) Did not complete primary school
( ) Completed primary school ( ) Completed Form 3
( ) Completed Form 5 ( ) Certificate
( ) A-Level/STPM/HSC ( ) Diploma

( ) First degree ( ) Advanced degree
( ) No answer ( ) Don't know
( ) Others (Specify : ____________

7. What is your current employment status??

( ) Government employee ( ) Private sector employee
( ) Self-employed ( ) Government retiree
( ) Private sector retiree ( ) Working while studying
( ) Still studying ( ) Housewife
( ) Unemployed ( ) Don't know
( ) No answer
( ) Others (Specify : ____________

8. What is your main occupation?

(for those currently working) (Please tick the correct category and write your position))

( ) Clerical support worker
( ) Technician and associate professional
( ) Service and sales worker
( ) Skilled agricultural, forestry and fishery worker
( ) Craft and related trades worker
( ) Plant and machine operator and assembler
( ) Elementary occupations
( ) Healthcare Occupations
( ) Profesional
( ) Manager
( ) Not applicable
( ) No answer
( ) Don't know

Please state your job position:____________________

9. What is your monthly family income??

( ) Below RM 2, 000 ( ) RM 2, 000 - RM 3, 000
( ) RM 3, 000 - RM 4, 000 ( ) RM 4, 000 - RM 5, 000
( ) RM 5, 000 - RM 10, 000 ( ) Above 10,000
( ) No answer ( ) Don't know

B. KNOWLEDGE ABOUT BREAST CANCER

This section aims to assess your level of knowledge about breast cancer. Please fill in the blank space below OR tick (✓) in the appropriate space.

Instructions : Please fill in the blank space below OR tick (✓) in the appropriate space.

10. There are many warning signs and symptoms of breast cancer. Please state as many as you know in the space below.

( ) No answer ( ) Don't know

The following may be **WARNING SIGNS** of breast cancer.

Instruction: Please tick (✓) in the space provided. Please tick one answer only..

No.	Question	Yes	No	No Answer	Don't Know
11.	In your opinion, could a change in the position of the nipple be a sign of breast cancer?				
12.	In your opinion, could a "nipple that seems pulled in" be a sign of breast cancer?				
13.	In your opinion, could "pain in one breast, or in the armpit" be a sign of breast cancer?				
14.	In your opinion, could "wrinkling or peeling skin around the breast" be a sign of breast cancer?				
15.	In your opinion, could "discharge or bleeding from the nipple" be a sign of breast cancer?				
16.	In your opinion, could a "lump or thickening of breast tissue" be a sign of breast cancer?				
17.	In your opinion, could a "rash on the nipple" be a sign of breast cancer?				
18.	In your opinion, could "redness on the skin of the breast" be a sign of breast cancer?				
19.	In your opinion, could a "lump or thickening of skin under the armpit" be a sign of breast cancer?				
20.	In your opinion, could a "change in the size of the breast or nipple" be a sign of breast cancer?				
21.	In your opinion, could a "change in the shape of the breast or nipple" be a sign of breast cancer?				

C. BREAST SELF-EXAMINATION

The next three questions are about checking for changes in your breasts.

Instruction: Please tick (✓) in the appropriate space. Please tick one answer only.
.

No.	Question	Rarely or never	At least once every 6 months	At least once a month	At least once a week	No Answer	Don't Know
22.	How often do you check your breasts?						

No.	Question	Not Confident at all	A little confident	Quite confident	Very confident	No Answer	Don't Know
23.	Are you confident you would notice a change in your breast?						

No.	Question	No	Yes	I have never noticed a change in one of my breasts	No Answer	Don't Know
24.	Have you ever seen a doctor about a change in one of your breasts?					

Instruction: Please fill in the blank or tick (✓) in the appropriate space.

25. If you noticed a change in your breast, how soon would you go to see a doctor??
Within _____________ weeks ( ) No answer ( ) Don't know

D. BARRIERS TO BREAST CANCER DETECTION

This section aims to find out what prevents you from seeing a doctor. Some patients delay seeing a doctor, even with symptoms they think might be serious.

Instruction: Please tick (✓) in the appropriate space. Please tick one answer only.

No.	Statement	No	Yes, always	Yes, sometimes	No answer	Don't Know
26.	I am too embarrassed to go see a doctor.					
27.	I am too scared to go see a doctor.					
28.	I worry that I would be wasting the doctor's time.					
29.	I find it difficult to talk to my doctor.					
30.	I find it difficult to make an appointment with a doctor.					
31.	I am too busy to make time to see a doctor.					
32.	There are too many other things for me to worry about.					
33.	I find it difficult to arrange transport to the clinic.					
34.	Worrying about what my doctor might find.					
35.	Not feeling confident to talk about my symptoms with a doctor.					

Instruction: Please fill in the blank or tick (✓) in the appropriate space.

36. Is there anything else that might stop you from going to see a doctor??

____

( ) No answer ( ) Don't know

E. KNOWLEDGE ABOUT BREAST CANCER RISKS

Instruction: Please tick (✓) in the appropriate space. Please tick one answer only.

		A woman aged 30	A woman aged 50	A woman aged 70	Women of any age	No Answer	Don't know
37.	In the next year, who is most likely to get breast cancer?						

No.	Questions	1 in 20 women	1 in 30 women	1 in 100 women	1 in 1000 women	No Answer	Don't know
38.	How many women do you think will get breast cancer in their lifetime?						

Instruction: Please fill in the blank or tick (✓) in the appropriate space.

39. There are many factors that might increase the chance of getting breast cancer. Please state as many as you can think of..

( ) No answer ( ) Don't know

The next questions are about what can increase the chance of getting breast cancer. To what extent do you agree that each of these things can increase the chance of getting breast cancer?

Instruction: Please tick (✓) in the appropriate space. Please tick one answer only..

No.	Risk Factor	Strongly disagree	Disagree	Not sure	Agree	Strongly agree	No answer
40.	Having a previous history of breast cancer						
41.	a) Using HRT (Hormone Replacement Therapy)						
	b) Using OCP (Oral Contraceptive Pills)						
42.	Drinking alcohol						
43.	Being overweight (BMI over 25)						
44.	Having a close relative with breast cancer						
45.	Giving birth at an older age or never having given birth ("Giving birth at an older age" means having the first child after the age of 30)						
46.	Starting periods at a young age ("young" means less than 12 years old)						
47.	Going through menopause at a late age ("late menopause" means menopause after the age of 55)						
48.	Doing moderate physical activity (i.e., less than 30 minutes a week, 5 times a week) ("moderate physical activity" means brisk walking, gardening, doing housework, active involvement in games and sports with children, walking with domestic pets, etc.))						

F. CLINICAL BREAST EXAMINATION

This section is about clinical breast examination to check whether you have breast cancer or not.

Instruction: Please tick (✓) in the appropriate space. Please tick one answer only

No.	Question	Yes	No	Not Sure	No Answer
49 (a).	Have you ever heard of a Clinical Breast Examination?				
49 (b).	In your lifetime, have your breasts ever been checked by a trained health professional like a doctor or nurse?				
49 (c).	Have your breasts been checked by a trained health professional like a doctor or nurse within the last year?				

49 (d). In the last 5 years, how many times was the examination done??
State the number: ______________ ( ) No Answer ( ) Don't know

49 (e). What is the distance from your residence to the clinic that performs Clinical Breast Examinations?
State the distance _______________km ( ) No Answer ( ) Don't know

49 (f). How many minutes does it take to get there?
State the duration ______________ min ( ) No Answer ( ) Don't know

Thank you for participating in this study..
