# Supplementary material for: Enhancing clinical breast examination (CBE) uptake: insights from women in northeastern Peninsular Malaysia
Source: PeerJ. 2026 Apr 6;14:e21029. doi: 10.7717/peerj.21029 (PMC13064672; doi:10.7717/peerj.21029)
Supplement: Supplemental Information 3 [file peerj-14-21029-s003.pdf]

No Siri:______

Kesedaran Kanser Payudara dan Faktor-Faktor Berkaitan Pemeriksaan Sendiri Payudara Serta Penerimaan Saringan Payudara dalam Kalangan Wanita di Kelantan

BORANG SOAL-SELIDIK

Borang soal-selidik ini mengandungi enam bahagian (A – F) seperti berikut:

Bahagian A	Maklumat sosiodemografi	9 soalan (1-9)
Bahagian B	Pengetahuan berkenaan kanser payudara	11 soalan (1-21)
Bahagian C	Pemeriksaan sendiri payudara	4 soalan (22-25)
Bahagian D	Halangan dalam melakukan pengesanan kanser payudara	11 soalan (26-36)
Bahagian E	Pengetahuan berkaitan risiko kanser payudara	12 soalan (37-48)
Bahagian F	Pemeriksaan klinikal payudara	5 soalan (49 a-e)

A. MAKLUMAT SOSIODEMOGRAFI

Bahagian ini bertujuan untuk mendapatkan maklumat berkaitan demografi anda.
Semua soalan perlu dijawab dan sila tandakan **(✓)** di dalam ruang yang berkenaan

1a. Apakah kewarganegaraan anda?

( ) Malaysia ( ) Bukan Malaysia
( ) Tiada jawapan ( ) Tidak tahu

1b. Umur: [ ] tahun

2. Apakah bangsa anda?

( ) Melayu ( ) Cina
( ) India ( ) Bumiputra lain
( ) Tiada jawapan ( ) Tidak tahu
( ) Lain-lain (Nyatakan) : ____________

3. Apakah agama anda?

( ) Islam ( ) Kristian
( ) Buddha ( ) Hindu
( ) Sikh ( ) Tidak tahu
( ) Tiada jawapan
( ) Lain-lain (Nyatakan) : ____________

4. Apakah status perkahwinan anda?

( ) Bujang ( ) Berkahwin
( ) Bercerai ( ) Janda/duda
( ) Tiada jawapan ( ) Tidak tahu

5. Berapakah bilangan ahli isi rumah anda? ______orang
(Semua orang yang tinggal di rumah, termasuk kanak- kanak)

( ) Tiada jawapan ( ) Tidak Tahu

6. Apakah taraf pendidikan anda yang paling tinggi?

( ) Tidak bersekolah ( ) Tidak tamat sekolah rendah
( ) Tamat sekolah rendah ( ) Tamat tingkatan 3
( ) Tamat tingkatan 5 ( ) Sijil
( ) A-Level/STPM/HSC ( ) Diploma
( ) Ijazah pertama ( ) Ijazah lanjutan
( ) Tiada jawapan ( ) Tidak tahu
( ) Lain-lain (Nyatakan) : _____________

7. Apakah status pekerjaan anda sekarang?

( ) Pekerja Kerajaan ( ) Pekerja swasta
( ) Bekerja sendiri ( ) Pesara kerajaan
( ) Pesara swasta ( ) Bekerja sambil belajar
( ) Masih belajar ( ) Surirumah
( ) Tiada pekerjaan ( ) Tidak tahu
( ) Tiada jawapan
( ) Lain-lain (Nyatakan) : _____________

8. Apakah pekerjaan utama anda? *(bagi orang yang sedang bekerja)*
(Sila tandakan kategori yang betul dan tuliskan jawatannya)

( ) Pekerja sokongan perkeranian
( ) Juruteknik dan profesional bersekutu
( ) Pekerja perkhidmatan dan jualan
( ) Pekerja mahir pertanian, perhutanan dan perikanan
( ) Pekerja kraf dan pekerja perdagangan berkaitan
( ) Loji dan operator mesin dan pemasang
( ) Pekerjaan asas
( ) Pekerjaan Penjagaan Kesihatan
( ) Profesional
( ) Pengurus
( ) Tidak berkaitan
( ) Tiada jawapan
( ) Tidak tahu

Sila nyatakan jawatan pekerjaan anda :_______________________

9. Apakah pendapatan bulanan keluarga anda?

( ) Bawah RM 2, 000 ( ) RM 2, 000 - RM 3, 000
( ) RM 3, 000 - RM 4, 000 ( ) RM 4, 000 - RM 5, 000
( ) RM 5, 000 - RM 10, 000 ( ) Atas 10,000
( ) Tiada jawapan ( ) Tidak tahu

B. PENGETAHUAN BERKENAAN KANSER PAYUDARA

Bahagian ini bertujuan untuk melihat tahap pengetahuan anda berkenaan kanser payudara

Arahan : Sila isi ruang kosong di bawah ATAU tandakan (✓) di dalam ruang yang berkenaan

10. Terdapat banyak tanda amaran dan gejala kanser payudara. Sila nyatakan seberapa banyak yang anda tahu dalam ruang kosong di bawah

( ) Tiada jawapan ( ) Tidak tahu

Berikut mungkin **TANDA AMARAN** kanser payudara.

Arahan : Sila tandakan (✓) di dalam ruang yang disediakan. Sila tanda satu jawapan sahaja.

		Ya	Tidak	Tiada Jawapan	Tidak Tahu
11.	Pada pendapat anda, mungkinkah **kelainan pada kedudukan puting** payudara adalah satu tanda kanser payudara?				
12.	Pada pendapat anda, mungkinkah "**puting macam tertarik ke dalam**" adalah satu tanda kanser payudara?				
13.	Pada pendapat anda, mungkinkah "**rasa sakit di salah satu payudara**, **atau di bahagian ketiak"** adalah satu tanda kanser payudara?				
14.	Pada pendapat anda, mungkinkah **"kedutan atau kulit mengelupas di sekitar payudara**" adalah satu tanda kanser payudara?				
15.	Pada pendapat anda, mungkinkah **"lelehan atau pendarahan keluar dari puting"** adalah satu tanda kanser payudara?				
16.	Pada pendapat anda, mungkinkah "**ketulan atau penebalan tisu payudara**" adalah satu tanda kanser payudara?				
17.	Pada pendapat anda, mungkinkah "**ruam di puting"** satu tanda kanser payudara?				
18.	Pada pendapat anda, mungkinkah "**kemerahan di kulit payudara**" satu tanda kanser payudara?				
19.	Pada pendapat anda, mungkinkah "**ketulan atau penebalan kulit di bawah ketiak**" satu tanda kanser payudara?				
20.	Pada pendapat anda, mungkinkah "**perubahan pada saiz payudara atau pada puting**" satu tanda kanser payudara?				
21.	Pada pendapat anda, mungkinkah **"perubahan pada bentuk payudara atau pada puting"** satu tanda kanser payudara?				

C. PEMERIKSAAN SENDIRI PAYUDARA

Tiga soalan berikutnya adalah tentang **mengesan perubahan pada payudara** anda.

Arahan : Sila tandakan (✓) di dalam ruang yang berkenaan. Sila tanda satu jawapan sahaja.

		Jarang atau tidak pernah	Sekurang-kurangnya sekali setiap 6 bulan	Sekurang-kurangnya sekali sebulan	Sekurang-kurangnya sekali seminggu	Tiada Jawapan	Tidak Tahu
22.	Berapa kerap anda memeriksa payudara anda?						

		Sama sekali tidak yakin	Sedikit yakin	Agak yakin	Sangat yakin	Tiada Jawapan	Tidak Tahu
23.	Adakah anda yakin anda akan tahu perubahan pada payudara anda?						

		Tidak	Ya	Tidak pernah perasan perubahan pada salah satu payudara saya	Tiada Jawapan	Tidak Tahu
24.	Pernahkah anda berjumpa doktor mengenai perubahan pada salah satu payudara anda?					

Soalan seterusnya adalah berkaitan **keinginan untuk mendapatkan bantuan.**

Arahan : Sila isi ruang kosong di bawah ATAU tandakan (✓) di dalam ruang yang berkenaan

25. Sekiranya anda mengesan perubahan pada payudara anda, berapa awal anda akan datang berjumpa doktor?
    Dalam tempoh______________ minggu ( ) Tiada jawapan ( ) Tidak tahu

D. HALANGAN DALAM MELAKUKAN PENGESANAN KANSER PAYUDARA

Bahagian ini bertujuan untuk mengetahui perkara yang **menghalang anda** daripada berjumpa doktor. Terdapat pesakit yang menangguhkan berjumpa doktor, walaupun mempunyai gejala yang mereka fikir mungkin serius.

Arahan : Sila tandakan (✓) di dalam ruang yang berkenaan. Sila tanda satu jawapan sahaja.

		Tidak	Ya, selalunya	Ya, kadang-kadang	Tiada Jawapan	Tidak Tahu
26.	Saya terlalu malu untuk pergi berjumpa doktor					
27.	Saya terlalu takut untuk pergi berjumpa doktor					
28.	Saya bimbang saya membuang masa doktor					
29.	Saya mendapati sukar untuk bercakap dengan doktor saya					
30.	Saya sukar membuat temujanji dengan doktor					
31.	Saya terlalu sibuk untuk meluangkan masa berjumpa doktor					
32.	Terlalu banyak perkara lain yang perlu saya bimbangkan					
33.	Saya sukar untuk menguruskan pengangkutan ke klinik					
34.	Bimbang tentang apa yang mungkin akan ditemui oleh doktor saya					
35.	Tidak merasa yakin untuk bercakap tentang gejala saya dengan doktor					

Arahan : Sila isi ruang kosong di bawah ATAU tandakan (✓) di dalam ruang yang berkenaan

36. Adakah perkara lain yang boleh menghalang anda pergi berjumpa doktor?

____

( ) Tiada jawapan ( ) Tidak tahu

E. PENGETAHUAN BERKAITAN RISIKO KANSER PAYUDARA

Arahan : Sila tandakan (✓) di dalam ruang yang berkenaan. Sila tanda satu jawapan sahaja.

Soalan berikut adalah mengenai siapa yang anda fikir berkemungkinan besar mendapat kanser payudara.

		Wanita berusia 30 tahun	Wanita berusia 50 tahun	Wanita berusia 70 tahun	Wanita dari sebarang peringkat usia	Tiada jawapan	Tidak tahu
37.	Dalam masa setahun ini, siapakah yang paling berkemungkinan mendapat kanser payudara?						

Soalan berikut adalah tentang berapa ramai wanita yang anda fikir akan menghidap kanser payudara dalam jangka hayat mereka.
Arahan : Sila tandakan (✓) di dalam ruang yang berkenaan. Sila tanda satu jawapan sahaja.

		**1 dalam 20 wanita**	**1 dalam 30 wanita**	**1 dalam 100 wanita**	**1 dalam 1000 wanita**	**Tiada jawapan**	**Tidak tahu**
38.	Berapa ramai wanita akan mengalami kanser payudara dalam jangka hayat mereka?						

Arahan : Sila isi ruang kosong di bawah ATAU tandakan (✓) di dalam ruang yang berkenaan

39. Terdapat banyak faktor yang mungkin meningkatkan kebarangkalian untuk mendapat kanser payudara.
Sila nyatakan seberapa banyak yang anda boleh fikirkan.

( ) Tiada jawapan ( ) Tidak tahu

Soalan seterusnya adalah mengenai apa yang boleh meningkatkan kebarangkalian mendapat kanser payudara. Sejauh manakah anda bersetuju bahawa setiap perkara ini boleh meningkatkan kebarangkalian mendapat kanser payudara.

Arahan : Sila tandakan (✓) di dalam ruang yang berkenaan. Sila tanda satu jawapan sahaja.

		**Sangat tidak setuju**	**Tidak setuju**	**Tidak pasti**	**Setuju**	**Sangat setuju**	**Tiada Jawapan**
40.	Mempunyai sejarah kanser payudara yang lalu						
41.	a) Penggunaan HRT (Terapi Penggantian Hormon)						
	b) Penggunaan OCP (Pil Perancang Keluarga)						
42.	Pengambilan alkohol						
43.	Berat badan berlebihan (BMI melebihi 25)						
44.	Mempunyai saudara terdekat yang menghidap kanser payudara						
45.	Melahirkan anak pada usia yang lanjut atau tiada pernah melahirkan anak *("Melahirkan anak pada usia yang lanjut" bermaksud melahirkan anak pertama pada usia melebihi 30 tahun)*						
46.	Datang haid pada usia yang muda *("muda" bermaksud kurang daripada 12 tahun)*						
47.	Putus haid pada usia yang lewat *("Putus haid lewat" bermaksud putus haid pada usia melebihi 55 tahun)*						
48.	Melakukan aktiviti fizikal sederhana (iaitu kurang daripada 30 minit seminggu, 5 kali seminggu) *("aktiviti fizikal sederhana" bermaksud berjalan pantas, berkebun, melakukan kerja rumah, penglibatan aktif dalam permainan dan sukan dengan kanak-kanak, berjalan dengan haiwan peliharaan domestik, dan sebagainya)*						

F. PEMERIKSAAN KLINIKAL PAYUDARA

Bahagian ini adalah berkaitan pemeriksaan klinikal payudara untuk memeriksa sama ada anda menghidap kanser payudara atau tidak.

Arahan : Sila tandakan (✓) di dalam ruang yang berkenaan. Sila tanda satu jawapan sahaja.

		**Ya**	**Tidak**	**Tidak pasti**	**Tiada Jawapan**
49 (a).	Pernahkah anda mendengar tentang Pemeriksaan Klinikal Payudara?				
49 (b).	Sepanjang hidup anda, adakah payudara anda pernah diperiksa oleh kakitangan kesihatan terlatih seperti doktor atau jururawat?				
49 (c).	Adakah payudara anda pernah diperiksa oleh kakitangan kesihatan terlatih seperti doktor atau jururawat **dalam masa satu tahun ini?**				

49 (d). Dalam tempoh 5 tahun lepas, berapa kali pemeriksaan tersebut dilakukan?
Nyatakan bilangan_______________ (   ) Tiada jawapan (   ) Tidak tahu

49 (e). Berapa jarak tempat kediaman anda dengan klinik yang menjalankan pemeriksaan Klinikal Payudara?
Nyatakan km___________________ (   ) Tiada jawapan (   ) Tidak tahu

49 (f). Berapa minit masa diperlukan untuk sampai ke sana?
Nyatakan minit_________________ (   ) Tiada jawapan (   ) Tidak tahu

Terima kasih atas kesudian anda menyertai kajian ini.
