# Supplementary material for: Enhancing clinical breast examination (CBE) uptake: insights from women in northeastern Peninsular Malaysia
Source: PeerJ. 2026 Apr 6;14:e21029. doi: 10.7717/peerj.21029 (PMC13064672; doi:10.7717/peerj.21029)
Supplement: Supplemental Information 4 [file peerj-14-21029-s004.docx]

**Enhancing Breast Cancer Screening Uptake: Insights from Women in Northeastern Peninsular Malaysia**

**CODEBOOK FOR SPSS RAW DATA**

|  | **SOCIODEMOGRAPHIC** |  |
| --- | --- | --- |
| 1. | NO. ID | NA |
| 2. | District (Name) | 1= Bachok, 2= Jeli, 3=Kota Bharu, 4=Kuala Krai, 5=Machang, 6= Pasir Mas, 7=Pasir Puteh, 8=Tanah Merah, 9=Tumpat |
| 3. | Age | Numerical |
| 4. | Citizenship | 1-Malaysia |
| 5. | Ethnicity | 1=Malay, 2=Chinese, 3=Indian |
| 6. | Religion | 1=Islam, 2=Christian, 3=Buddha, 4=Hindu |
| 7. | Marital Status | 1=Single, 2=Married, 3= Divorced, 4=Widow, 99=Unknown |
| 8. | House Members | Numerical |
| 9. | Education Status | 1=No Formal Education, 2= Incomplete Primary School, 3= Completed Primary School, 4= Completed Form 3, 5=Completed Form 5, 6= Certificate, 7=A-level, STPM, 8=Diploma, 9=First Degree. |
| 10. | Employment Status | 1=Government Servant, 2=Private Sector, 3=Self-employed, 4=Government Pensioner, 5=Private Pensioner, 6=Part-time employee, 7=Student, 8=Housewife, 9=Unemployed, 98= No Answer, 99=Don’t Know |
| 11. | Occupation | 1=Manager, 2=Professional, 3=Technician and Allied Profession, 4=Clerk and support staff, 5=Sales and service employee, 6=Skilled workers in agriculture, forestry and fisheries, 7=Craft workers and related trade workers, 8=Plant and machine and installation operators, 9=Elementary occupation, 10=Healthcare provider, 97=Not Related, 98=No Answer, 99=Don’t know |
| 12. | Real Occupation | Not Aplicable |
| 13. | Household Income | 1=Below RM2000, 2=RM2000-RM2999, 3=RM3000-RM3999, 4=RM4000-RM4999, 5=RM5000-RM9999, 6=RM10000 and Above, 98=No Answer, 99= Don’t Know |
|  | **Aware of Breast Cancer Signs and symptoms** |  |
| 14. | Breast position | 1= Yes, 2=No, 98=No Answer, 99=Don’t Know |
| 15. | Breast puling | 1= Yes, 2=No, 98=No Answer, 99=Don’t Know |
| 16. | Breast pain | 1= Yes, 2=No, 98=No Answer, 99=Don’t Know |
| 17. | Breast pucker | 1= Yes, 2=No, 98=No Answer, 99=Don’t Know |
| 18. | Breast Discharge | 1= Yes, 2=No, 98=No Answer, 99=Don’t Know |
| 19. | Breast lump | 1= Yes, 2=No, 98=No Answer, 99=Don’t Know |
| 20. | Breast rash | 1= Yes, 2=No, 98=No Answer, 99=Don’t Know |
| 21. | Breast redness | 1= Yes, 2=No, 98=No Answer, 99=Don’t Know |
| 22. | Breast arm lump | 1= Yes, 2=No, 98=No Answer, 99=Don’t Know |
| 23. | Breast size | 1= Yes, 2=No, 98=No Answer, 99=Don’t Know |
| 24. | Breast shape | 1= Yes, 2=No, 98=No Answer, 99=Don’t Know |
|  | **Breast self-examination** |  |
| 25. | BSE Uptake | 1=Rarely or never, 2=At least once every 6 months, 3=At least once a month, 4=At least once a week, 98=No Answer, 99=Don’t know |
| 26. | Breast confidence | 1=Not at all confident, 2= Not very confident, 3= Fairly confident, 4=Very Confident, 98=No Answer, 99=Don’t Know |
| 27. | Seeing Doctor | 1=No, 2=Yes, 3=Never noticed a change in one of my breast, 98=No Answer, 99=Don’t Know |
| 28. | Healthcare seeking time | Numerical |
|  | **Barriers to cancer detection** |  |
| 29. | Shy | 1=No, 2=Yes, always, 3= Yes, sometimes, 98=No Answer, 99=Don’t Know |
| 30. | Fear | 1=No, 2=Yes, always, 3= Yes, sometimes, 98=No Answer, 99=Don’t Know |
| 31. | Time wasting | 1=No, 2=Yes, always, 3= Yes, sometimes, 98=No Answer, 99=Don’t Know |
| 32. | Difficult to talk to | 1=No, 2=Yes, always, 3= Yes, sometimes, 98=No Answer, 99=Don’t Know |
| 33. | Difficult appointment | 1=No, 2=Yes, always, 3= Yes, sometimes, 98=No Answer, 99=Don’t Know |
| 34. | Busy | 1=No, 2=Yes, always, 3= Yes, sometimes, 98=No Answer, 99=Don’t Know |
| 35. | Too worry | 1=No, 2=Yes, always, 3= Yes, sometimes, 98=No Answer, 99=Don’t Know |
| 36. | Transportation | 1=No, 2=Yes, always, 3= Yes, sometimes, 98=No Answer, 99=Don’t Know |
| 37. | Fear of result | 1=No, 2=Yes, always, 3= Yes, sometimes, 98=No Answer, 99=Don’t Know |
| 38. | Not confidence | 1=No, 2=Yes, always, 3= Yes, sometimes, 98=No Answer, 99=Don’t Know |
| 39. | Age factor | 1=A 30 year old woman, 2=A 50 year old woman, 3=A 70 year old woman, 4=A woman of any age, 98=No Answer, 99=Don’t Know |
| 40. | Breast cancer possibility | 1=1 in 20 women, 2=1 in 30 women, 3=1 in 100 women, 1 in 1000 women, 98=No Answer, 99=Don’t Know |
|  | **Aware of Breast Cancer Risk** |  |
| 41. | Past history | 1=Strongly disagree, 2=disagree, 3=Not sure, 4=Agree, 5=Strongly Agree, 98=No Answer |
| 42. | HRT | 1=Strongly disagree, 2=disagree, 3=Not sure, 4=Agree, 5=Strongly Agree, 98=No Answer |
| 43. | OCP | 1=Strongly disagree, 2=disagree, 3=Not sure, 4=Agree, 5=Strongly Agree, 98=No Answer |
| 44. | Alcohol | 1=Strongly disagree, 2=disagree, 3=Not sure, 4=Agree, 5=Strongly Agree, 98=No Answer |
| 45. | BMI | 1=Strongly disagree, 2=disagree, 3=Not sure, 4=Agree, 5=Strongly Agree, 98=No Answer |
| 46. | Family history | 1=Strongly disagree, 2=disagree, 3=Not sure, 4=Agree, 5=Strongly Agree, 98=No Answer |
| 47. | No kids | 1=Strongly disagree, 2=disagree, 3=Not sure, 4=Agree, 5=Strongly Agree, 98=No Answer |
| 48. | Periods | 1=Strongly disagree, 2=disagree, 3=Not sure, 4=Agree, 5=Strongly Agree, 98=No Answer |
| 49. | Menopause | 1=Strongly disagree, 2=disagree, 3=Not sure, 4=Agree, 5=Strongly Agree, 98=No Answer |
| 50. | Exercise | 1=Strongly disagree, 2=disagree, 3=Not sure, 4=Agree, 5=Strongly Agree, 98=No Answer |
|  | **CBE** |  |
| 51. | Aware of CBE | 1=No, 2=Yes, 98=No Answer, 99=Don’t Know |
| 52. | Ever done CBE | 1=No, 2=Yes, 98=No Answer, 99=Don’t Know |
| 53. | Done CBE in this 1 year | 1=No, 2=Yes, 98=No Answer, 99=Don’t Know |
| 54. | CBE clinic distance | Numerical |
| 55. | **Recategorize for Sociodemographic** |  |
| 56. | Household income Result | 1=No Permanent income, 2= <RM3000, 3= RM3000-RM4999, 4=RM5000 and above |
| 57. | Marital status Result | 1=Single, 2=Married, 3= Divorced/Widow |
| 58. | Education level Result | 1-Primary and Secondary school, 2= Certificate/Malaysia Higher Education certification/diploma, 3= Degree and above |
|  | Age Result | 0= Less than 40 years old, 1=40 years old and above |
| 59. | Employment status Result | 1= Others, student, pensioner, unemployed, 2=Government sector, 3= Private sector, 4=Housewife |
| 60. | House member result | 0=1-2, 1=3-5, 2=6 and above |
|  | **Recategorize for awareness of Breast** **cancer signs and symptoms** |  |
| 61. | Breast position | 0=No, 1=Yes |
| 62. | Breast puling | 0=No, 1=Yes |
| 63. | Breast pain | 0=No, 1=Yes |
| 64. | Breast pucker | 0=No, 1=Yes |
| 65. | Breast Discharge | 0=No, 1=Yes |
| 66. | Breast rash | 0=No, 1=Yes |
| 67. | Breast redness | 0=No, 1=Yes |
| 68. | Breast size | 0=No, 1=Yes |
| 69. | Breast shape | 0=No, 1=Yes |
| 70. | Breast lump | 0=No, 1=Yes |
| 71. | Breast arm lump | 0=No, 1=Yes |
| 72. | Sum of signs and symptoms | Numerical |
| 73. | Result aware of S&S | 0=Not aware, 1=aware |
|  | **Breast Self-Examination** |  |
| 74. | BSE Uptake result | 0=No, 1=Yes |
| 75. | Confidence | 0=No, 1=Yes |
| 76. | Seeing Doctor | 0=No, 1=Yes |
| 77. | Duration of healthcare seeking |  |
|  | **Barrier Recategorize** |  |
| 78. | Barrier shy | 0=No, 1= No answer or don’t know, 2=Yes sometimes, 3=Yes always |
| 79. | Barrier fear | 0=No, 1= No answer or don’t know, 2=Yes sometimes, 3=Yes always |
| 80. | Barrier wasting time | 0=No, 1= No answer or don’t know, 2=Yes sometimes, 3=Yes always |
| 81. | Barrier difficult to talk | 0=No, 1= No answer or don’t know, 2=Yes sometimes, 3=Yes always |
| 82. | Barrier appt difficulty | 0=No, 1= No answer or don’t know, 2=Yes sometimes, 3=Yes always |
| 83. | Barrier busy | 0=No, 1= No answer or don’t know, 2=Yes sometimes, 3=Yes always |
| 84. | Barrier anxious | 0=No, 1= No answer or don’t know, 2=Yes sometimes, 3=Yes always |
| 85. | Barrier transportation | 0=No, 1= No answer or don’t know, 2=Yes sometimes, 3=Yes always |
| 86. | Barrier lack of confidence | 0=No, 1= No answer or don’t know, 2=Yes sometimes, 3=Yes always |
| 87. | Barrier fear of diagnosis | 0=No, 1= No answer or don’t know, 2=Yes sometimes, 3=Yes always |
| 88. | Barrier SUM | Numerical |
| 89. | Perceived barrier result | 0= Has barrier, 1=No barrier |
| 90. | Age risk factor result | 0=No, 1=Yes |
| 91. | Breast cancer Possibility | 0=No, 1=Yes |
|  | **Risk Factor recategorize** |  |
| 92. | Past history | 0=Strongly Disagree, 1=Disagree, 2= Not sure or No Answer, 3=Agree, Strongly Agree |
| 93. | HRT | 0=Strongly Disagree, 1=Disagree, 2= Not sure or No Answer, 3=Agree, Strongly Agree |
| 94. | OCP | 0=Strongly Disagree, 1=Disagree, 2= Not sure or No Answer, 3=Agree, Strongly Agree |
| 95. | Alcohol | 0=Strongly Disagree, 1=Disagree, 2= Not sure or No Answer, 3=Agree, Strongly Agree |
| 96. | BMI | 0=Strongly Disagree, 1=Disagree, 2= Not sure or No Answer, 3=Agree, Strongly Agree |
| 97. | Family history | 0=Strongly Disagree, 1=Disagree, 2= Not sure or No Answer, 3=Agree, Strongly Agree |
| 98. | No kids | 0=Strongly Disagree, 1=Disagree, 2= Not sure or No Answer, 3=Agree, Strongly Agree |
| 99. | Early menses | 0=Strongly Disagree, 1=Disagree, 2= Not sure or No Answer, 3=Agree, Strongly Agree |
| 100. | Menopause | 0=Strongly Disagree, 1=Disagree, 2= Not sure or No Answer, 3=Agree, Strongly Agree |
| 101. | Exercise | 0=Strongly Disagree, 1=Disagree, 2= Not sure or No Answer, 3=Agree, Strongly Agree |
| 102. | Sum aware of risk factor | Numerical |
| 103. | Aware of risk factor result | 0=Not aware, 1=Aware |
| 104. | Aware CBE | 0=No, 1=Yes |
| 105. | CBE uptake | 0=No, 1=Yes |
| 106. | CBE in 1 year | 0=No, 1=Yes |
| 107. | CBE in 5 years | Numerical |
| 108. | Accessibility to CBE | 0= >5Km, 1= 5km and below |
|  |  |  |
|  |  |  |
|  |  |  |
|  |  |  |
|  |  |  |
|  |  |  |
|  |  |  |
|  |  |  |
|  |  |  |
|  |  |  |
|  |  |  |
|  |  |  |
|  |  |  |
|  |  |  |
|  |  |  |
|  |  |  |
|  |  |  |
